# Prolonged Survival of Acute Lymphoblastic Leukemia with Intrathecal Treatments for Isolated Central Nervous System Relapse

**DOI:** 10.1155/2018/8765285

**Published:** 2018-01-31

**Authors:** Elan Gorshein, Sheila Kalathil, Mecide Gharibo

**Affiliations:** ^1^Rutgers Robert Wood Johnson Medical School, 3rd Floor, New Brunswick, NJ, USA; ^2^Hackensack Meridian Health Jersey Shore University, 1945 Route 33, Ackerman Building, 3rd Floor, Neptune, NJ 07753, USA; ^3^Bayer U.S. LLC, 100 Bayer Boulevard, Whippany, NJ 07891-0915, USA

## Abstract

Acute lymphoblastic leukemia is commonly cured when diagnosed in the pediatric population. It portends a poorer prognosis if present in adult patients. Although adults frequently achieve complete remission, relapse rates are substantial, particularly among the elderly and high-risk populations. In the absence of prophylactic intrathecal chemotherapy, more than half of patients may develop CNS involvement or relapse, which is associated with significant risk for systemic illness. This report describes a patient with acute lymphoblastic leukemia with repeated isolated CNS relapses. This case should remind clinicians that isolated CNS disease in the absence of systemic recurrence could successfully respond to intrathecal therapy and offer patients a favorable quality of life.

## 1. Introduction

Despite the overall very poor prognosis of isolated CNS relapse in acute lymphoblastic leukemia, long-term remission (in the absence of systemic relapse) may be obtained with intrathecal chemotherapy. We present a patient case that has maintained an excellent quality of life for more than eight years in the setting of greater than eighty intrathecal treatments. Limited data exist in regard to prolonged intrathecal courses in acute lymphoblastic leukemia patients, and, to our knowledge, this is the first description of successful treatment for isolated CNS relapse in the absence of systemic recurrence in this disease.

## 2. Case Presentation

The patient is a 70-year-old male with a past medical history of hypertension and dyslipidemia, who presented in September 2009 with progressive fatigue. He was noted to have a white blood cell count of 3.3 thousand/ul, with 22% circulating blasts. His staging bone marrow biopsy showed a hypercellular marrow, 95% cellularity with complete replacement by lymphoid blasts, with flow study detecting 84% blasts expressing dim CD45 with dim TdT, CD19, CD10, CD79A, CD22, HLA-DR, and variable intensity for CD20, and CD34. The diagnosis was consistent with pre-B acute lymphoblastic leukemia, and the FISH study was negative for BCR-ABL, as well as 11Q23 translocation. His initial cytogenetic study showed a normal male karyotype. Incidentally, he was noted to have a small subacute frontoparietal subdural hematoma. Initial CSF evaluation was negative for leukemic blasts.

Our patient began induction therapy on CALGB-9111 protocol. He was noted to have persistent disease after the initial induction therapy and was transitioned to Hyper-CVAD part B with rituximab. He received intrathecal chemotherapy with methotrexate and was noted to have CNS leukemia involvement in November 2009. A recovery bone marrow biopsy in December 2009 showed complete remission, with adequate cellularity and normal trilineage hematopoiesis without residual leukemia, and normal male karyotype. He began four weekly intrathecal therapy sessions for CNS leukemia. Repeat CSF cytology studies were negative. He was deemed a poor candidate for allogeneic stem cell transplantation secondary to his age and comorbidities. In January 2010, the patient began an R-CHOP regimen, as he experienced significant complications from the Hyper-CVAD therapy (*Stenotrophomonas maltophilia* bacteremia with neutropenic fevers). A bone marrow biopsy after four cycles of R-CHOP showed a regenerative marrow with no evidence of residual leukemia and normal male karyotype. He then received one year of maintenance treatment with R-POMP and intrathecal chemotherapy, at which point a bone marrow biopsy in April 2011 showed no evidence of leukemia.

CSF studies at that time showed no leukemia. He then was started on maintenance therapy with rituximab therapy every two months for six cycles and intrathecal therapy every four months. He completed the course of treatment in February 2012. He had stable peripheral blood counts with no evidence of relapsed disease noted with ongoing CR1 status. In March 2013, he experienced proximal muscle weakness. MRI of his cervical, thoracic, and lumbar spine revealed no evidence of disease involvement. A diagnostic lumbar puncture for evaluation of CNS leukemia confirmed relapsed CNS disease, where the CSF fluid demonstrated a glucose level of less than 2 mg/with over 2700 nucleated cells, the majority of which were blasts consistent with pre-B acute lymphoblastic leukemia, expressing CD19, CD20, CD10, TDT, and CD34. A subset of the cells appeared to express dim CD2 with no other apparent antigen expression noted. The flow study detected 98% of the cells were blasts. An MRI showed no leptomeningeal involvement, and a bone marrow biopsy was negative. He had an Ommaya reservoir placed in preparation for long-term intrathecal treatments.

A complete history of the patient's intrathecal treatments is listed in Table 1. He began serial intrathecal chemotherapy alternating with methotrexate and cytarabine weekly followed by every two weeks. The patient was noted to have clearance of leukemia blasts, so the interval of intrathecal treatment was extended to every 2-3 months with alternating methotrexate and cytarabine. Repeat CSF cytology was negative throughout the sessions until April 2015, when an isolated CNS relapse was noted with no clinical signs or symptoms of systemic relapse. At that point, the patient's intrathecal chemotherapy interval was shortened to weekly therapy. CSF studies revealed no disease involvement. However, as treatment was prolonged to monthly administration, recurrent isolated CNS relapses were observed. The patient once again cleared the CSF cytology with more frequent intrathecal chemotherapy. An MRI of the brain in December 2015 showed no leptomeningeal disease. In September 2016, CSF cytology confirmed relapsed disease, but peripheral counts remained normal. His chemotherapy at that time remained with cytarabine and alternating methotrexate. He was transitioned to thiotepa in March 2017 following positive cytology. His repeat CSF studies were negative for leukemia, but in April 2017, the patient experienced another episode of isolated CNS relapse. He was then switched to triple therapy with intrathecal hydrocortisone, methotrexate, and cytarabine. He thereafter also received intrathecal rituximab. During these courses of treatment, CSF studies were intermittently positive for disease recurrence without signs or symptoms of systemic disease, and interval peripheral flow cytometry has demonstrated no diagnostic evidence of circulating B-cell lymphoblastic leukemia. Clinically, the patient is maintaining his quality of life eight years after his initial diagnosis, with no focal neurologic symptoms or side effects from the intrathecal treatments and no evidence of systemic relapse to date.

## 3. Discussion

Acute lymphoblastic leukemia most commonly occurs in the pediatric population. However, it is responsible for 20% of adult leukemia cases and carries a significantly poorer prognosis than in children, with overall five-year survival rates of 30–40% [1, 2]. This is attributed to increased drug resistance, higher risk leukemia, medical comorbidities, and poorer treatment tolerance and compliance [3]. Among adults older than 55–60 years, which defines high-risk disease, the probability of survival decreases to 20% at three years [4, 5]. With standard intensive induction chemotherapy, approximately 85–90% of adult patients will achieve a complete remission [6]. For elderly patients, CR rates are approximately 55–60% [7–9].

CNS involvement at the time of acute lymphoblastic leukemia diagnosis is noted in about 6% of patients [10]. The rate of detection of CNS disease is maximized by including cytological evaluation with flow cytometry [11, 12]. In the absence of CNS prophylaxis, as many as 50–75% of patients may develop CNS disease [13]. For those who receive systematic preventive therapy, CNS relapse occurs in 2–10% of patients [14]. With systemic relapse, CNS leukemia becomes more common. However, the likelihood of an isolated CNS relapse following a remission from acute lymphoblastic leukemia is approximately 5% with standard treatment [15]. For these patients, median overall survival is approximately 6 months, and systemic recurrence risk is high and typically within a few months [15].

Our patient manifested disease control in the absence of systemic recurrence with the administration of intrathecal rituximab chemotherapy. Intravenous rituximab treatment achieves CNS concentrations that are significantly lower than intrathecal dosing [16]. However, there is limited data discussing the potential role of intrathecal rituximab in acute lymphoblastic leukemia with CNS relapse. In a case series of acute lymphoblastic leukemia with CNS relapse disease, five of seven patients demonstrated a complete remission following two years of CSF fluid analysis [17]. However, these patients received prior spinal or cranial irradiation and were significantly younger (none older than age 21) than our patient [17]. More recently, a case series of pediatric patients with B-cell lymphoid CD20+ malignancies reported an ongoing complete remission in only one of three patients with acute lymphoblastic leukemia who were treated with intraventricular or intrathecal rituximab [18]. Thus, preliminary data on the use of intrathecal or intraventricular rituximab therapy for CNS involvement of CD20+ acute lymphoblastic leukemia suggest a potential benefit, but requires further evaluation in this setting.

## Figures and Tables

**Table 1:** Chemotherapy number of IT treatments total dose (mg).

Methotrexate 10 mg	2	20 mg
Methotrexate 12 mg	45	540 mg
Methotrexate 15 mg	6	90 mg
Cytarabine 20 mg	2	40 mg
Cytarabine 30 mg	4	120 mg
Cytarabine liposomal 50 mg	1	50 mg
Cytarabine 70 mg	14	980 mg
Cytarabine 75 mg	2	150 mg
Cytarabine 100 mg	4	400 mg
Thiotepa 10 mg	3	30 mg
Hydrocortisone 15 mg	9	135 mg
Rituximab 10 mg	2	20 mg
Rituximab 25 mg	5	125 mg

Intrathecal chemotherapy received.
